# A205 EVALUATING THE ASSOCIATION BETWEEN PERIPHERAL BLOOD EOSINOPHILS AND DRUG RESPONSE IN CROHN'S DISEASE: CONTINUING ANALYSIS

**DOI:** 10.1093/jcag/gwac036.205

**Published:** 2023-03-07

**Authors:** B Cai, A Wilson

**Affiliations:** Gastroenterology, Western University, London, Canada

## Abstract

**Background:**

Th2 cytokines, IL-5 and IL-13 enhance peripheral and mucosal eosinophil survival, recruitment and degranulation, facilitating inflammation in Crohn's Disease. In a preliminary analysis, peripheral eosinophilia (PBE) is seen to have an association with rates of steroid response and anti-TNF response in CD patients. Participants with high PBE (> 200 cells/μL) appear to be more steroid-responsive but less responsive to Th1-targeting anti-TNF therapies. We hypothesize the pattern of PBE at CD diagnosis can help identify distinct subsets within a larger CD population and correlate with response to treatments such as prednisone or anti-TNFs.

**Purpose:**

We aim to evaluate the pattern of PBE of CD patients at time of diagnosis (prior to drug exposure) and with each subsequent treatment; and if baseline PBE or any changes seen with drug exposures are predictive of treatment response.

**Method:**

A retrospective cohort study is ongoing with CD patients exposed to glucocorticoids and an anti-TNF seen at 3 hospitals affiliated with University of Western Ontario. Patients were identified using administrative databases and reviewed for biochemical data (complete blood count) and disease activity (Harvey Bradshaw Index) at baseline, before and after each drug exposure. Participants were classified as having high PBE (eosinophils>200 cells/μL) versus low PBE (eosinophils <200 cells/μL). To date, 350 patients have been screened. Subgroup analyses of PBE > 300 cells/μL, and differences between female and male patients will be carried out.

**Result(s):**

46 of 200 CD patients are included in the continuing analysis with a mean age of 45 years. 26 had PBE >200 cells/μL at baseline and 20 did not. The median number therapies used was 4 (IQR=0.75). All received glucocorticoids followed by an anti-TNF. There was no difference in the occurrence of hospitalization or surgery between the two groups. Overall 50% participants with high PBE >200 and >300 cells/μL had clinical response to glucocorticoid exposure, seen as a 3-point decrease in HBI compared to 45%, 47% in the low PBE cohort (n=13/26 vs. n=9/20 p=0.77; n=6/12 vs. n=16/34 p=1.0 respectively). With subsequent anti-TNF exposure, PBE rebounded in 7 participants. 36% patients in the high PBE group required anti-TNF dose escalation versus 24% in the low PBE group (n=9/25 vs. n=5/21, p=0.52). The proportion of patients with anti-TNF discontinuation was similar in both groups (high PBE 19.2%, n=5/26 vs. low PBE 15%, n=3/20, p=1.0). Men had higher steroid response rates compared to women in both high and low PBE groups (n=6/8 vs. n=8/18 p=0.21; n=4/9 vs. 4/11 p=1.0 respectively).

**Conclusion(s):**

Peripheral eosinophilia is seen in varying degrees in CD patients. Participants with high PBE are more steroid-responsive. High PBE patients overall were less responsive to anti-TNF therapies, requiring more dose-escalation and discontinued anti-TNF treatment. Completion of this study will help clarify the association between PBE in CD and treatment response.

**Please acknowledge all funding agencies by checking the applicable boxes below:**

None

**Disclosure of Interest:**

None Declared

